# Soil phosphorus heterogeneity promotes tree species diversity and phylogenetic clustering in a tropical seasonal rainforest

**DOI:** 10.1002/ece3.2529

**Published:** 2016-11-16

**Authors:** Wumei Xu, Xiuqin Ci, Caiyun Song, Tianhua He, Wenfu Zhang, Qiaoming Li, Jie Li

**Affiliations:** ^1^ Plant Phylogenetics and Conservation GroupCenter for Integrative ConservationXishuangbanna Tropical Botanical GardenChinese Academy of SciencesKunmingYunnanChina; ^2^ University of Chinese Academy of SciencesBeijingChina; ^3^ Department of Environment and AgricultureCurtin UniversityPerthWAAustralia; ^4^ Key Laboratory of Tropical Forest EcologyXishuangbanna Tropical Botanical GardenChinese Academy of SciencesMenglunYunnanChina

**Keywords:** community phylogenetic structure, heterogeneity, nitrogen, phosphorus, species diversity, tropical forest

## Abstract

The niche theory predicts that environmental heterogeneity and species diversity are positively correlated in tropical forests, whereas the neutral theory suggests that stochastic processes are more important in determining species diversity. This study sought to investigate the effects of soil nutrient (nitrogen and phosphorus) heterogeneity on tree species diversity in the Xishuangbanna tropical seasonal rainforest in southwestern China. Thirty‐nine plots of 400 m^2^ (20 × 20 m) were randomly located in the Xishuangbanna tropical seasonal rainforest. Within each plot, soil nutrient (nitrogen and phosphorus) availability and heterogeneity, tree species diversity, and community phylogenetic structure were measured. Soil phosphorus heterogeneity and tree species diversity in each plot were positively correlated, while phosphorus availability and tree species diversity were not. The trees in plots with low soil phosphorus heterogeneity were phylogenetically overdispersed, while the phylogenetic structure of trees within the plots became clustered as heterogeneity increased. Neither nitrogen availability nor its heterogeneity was correlated to tree species diversity or the phylogenetic structure of trees within the plots. The interspecific competition in the forest plots with low soil phosphorus heterogeneity could lead to an overdispersed community. However, as heterogeneity increase, more closely related species may be able to coexist together and lead to a clustered community. Our results indicate that soil phosphorus heterogeneity significantly affects tree diversity in the Xishuangbanna tropical seasonal rainforest, suggesting that deterministic processes are dominant in this tropical forest assembly.

## 1 Introduction

**Figure 1:**
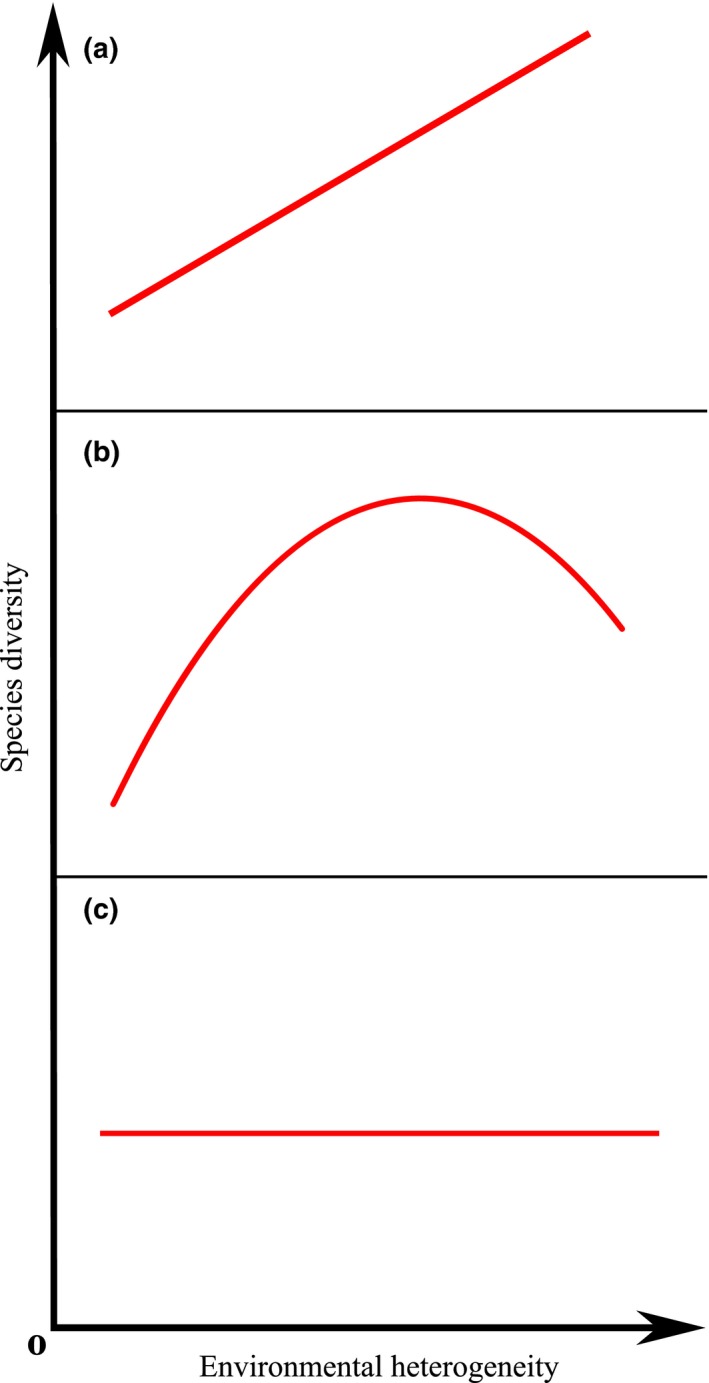
Theoretical predictions between environmental heterogeneity and species diversity. (a) Classical niche theory. Environmental heterogeneity and niche partitioning as the main factors structuring ecological communities and promoting species coexistence. (b) General unimodal theory. Heterogeneity increases, while the amount of effective area available for individual species decreases, reducing population sizes and increasing the likelihood of stochastic extinctions. (c) Neutral community ecology theory. Stochastic processes dominate the community assembly, and the species diversity within the community is not related to environmental heterogeneity

The tropical forests harbor an enormous diversity of plant species and are conservation priorities in a fast‐changing world (Figure S1); however, the mechanisms that determine tropical tree community assembly remain important yet not well‐solved questions in community ecology (Valladares, Bastias, Godoy, Granda, & Escudero, 2015). The classical niche theory predicts that communities with more environmental heterogeneity will have higher species diversity than those with less heterogeneity because more niches can be partitioned in a heterogeneous habitat (Figure 1a; Hutchinson, 1957; Kadmon & Allouche, 2007; Macarthur & Macarthur, 1961; Ricklefs, 1977; Svenning, 2001). Environmental heterogeneity is typically determined to be the universal driver of species diversity in a variety of ecosystems (Stein, Gerstner, & Kreft, 2014), for example, limestone pavement (Lundholm & Larson, 2003), pine forest (Gundale, Metlen, Fiedler, & DeLuca, 2006), and temperate swamp forest (Douda, Doudova‐Kochankova, Boublik, & Drasnarova, 2012) ecosystems. However, in the tropics, the demonstration of a positive correlation between environmental heterogeneity and species diversity is still lacking (Holl, Stout, Reid, & Zahawi, 2013). First, resource heterogeneity usually covaries with average resource supply rate, making the effect of heterogeneity difficult to separate (Lundholm, 2009; Stevens & Carson, 2002). Second, the general unimodal theory predicts a unimodal relationship rather than a positive correlation between environmental heterogeneity and species diversity (Figure 1b) because as heterogeneity increases, the effective area available for individual species decreases, reducing population sizes and increasing the likelihood of stochastic extinctions (Allouche, Kalyuzhny, Moreno‐Rueda, Pizarro, & Kadmon, 2012; Kadmon & Allouche, 2007). Additionally, neutral community ecology theory suggests that stochastic processes (e.g., dispersal limitations or ecological drift) are dominant in regulating plant distributions in the tropics; thus, no correlation between heterogeneity and diversity would be expected within such a community (Figure 1c; Hubbell, 2001; Rosindell, Hubbell, & Etienne, 2011). Although the neutral theory provides new insights into how tropical forests are structured, the strict assumption of ecological equivalence among species has limited empirical support in general (Chave, 2004; Gaston & Chown, 2005). To date, several studies have indicated that deterministic rather than the neutral processes are dominant in tropical forest assembly (Brown et al., 2013; Condit, Engelbrecht, Pino, Perez, & Turner, 2013; John et al., 2007; Kraft, Valencia, & Ackerly, 2008; Yang et al., 2014, 2015).

If deterministic processes dominate the assembly of Xishuangbanna tropical seasonal rainforest (Yang et al., 2014, 2015), a positive correlation between environmental heterogeneity and species diversity could be expected. However, direct evidence for a positive heterogeneity–diversity relationship within this area is lacking, and how environmental heterogeneity affects plant diversity within the tropics is largely unknown. Recent studies on phylogenetic community ecology have provided new insights into environment–plant interactions and how communities are assembled (Cavender‐Bares, Kozak, Fine, & Kembel, 2009; Qian & Jiang, 2014; Webb, Ackerly, McPeek, & Donoghue, 2002). For example, Stevens, Gavilanez, Tello, and Ray (2012) have found that an increase in environmental heterogeneity (food heterogeneity) significantly affects rodent species diversity within a desert ecosystem, and on the basis of further community phylogenetic analysis, that the species in the community are phylogenetically overdispersed in environments with low heterogeneity and tend to cluster with an increase in environmental heterogeneity. Therefore, with an increase in environmental heterogeneity, closely related and similar species could coexist within the community.

Nitrogen (N) and phosphorus (P) are generally considered the two most limiting elements to terrestrial vegetation and play an essential role in plant community assembly (Daufresne & Hedin, 2005; Reich & Oleksyn, 2004). In tropical ecosystems, phosphorus is usually suggested as the most limiting soil nutrient (Laliberte et al., 2013; Vitousek, 1984; Vitousek, Porder, Houlton, & Chadwick, 2010; Vitousek & Sanford, 1986). A previous study in Xishuangbanna tropical seasonal rainforest has confirmed that soil phosphorous is very deficient and substantially affects the community assembly (Xu et al., 2016). In this study, we randomly established 39 forest vegetation plots each with 400 m^2^ (20 × 20 m) in the Xishuangbanna tropical seasonal rainforest in southwestern China (Figure S2). Soil nutrient (N and P) availability and heterogeneity, tree species diversity, and community phylogenetic structure were measured in each plot. We attempted to explore the following two questions: (1) Is the soil N or P heterogeneity correlated with tree species diversity in a community? (2) What is the mechanism underlying the relationship between soil heterogeneity and species diversity within a community?

## 2 Methods

### 2.1 Study site

Thirty‐nine plots each with 400 m^2^ (20 × 20 m) were randomly established in an area of approximately 100 km^2^ in the Xishuangbanna tropical seasonal rainforest in southwestern China (Figures S1 and S2). This region has an average annual rainfall of 1,493 mm, and laterite soils developed from siliceous rocks (Cao, Zou, Warren, & Zhu, 2006). The region is part of the Indo‐Burma biodiversity hotspot (Myers, Mittermeier, Mittermeier, da Fonseca, & Kent, 2000). The dominant tree species in the plots included *Pittosporopsis kerrii* Craib (Icacinaceae)*, Parashorea chinensis* H. Wang (Dipterocarpaceae)*,* and *Garcinia cowa* Roxburgh (Clusiaceae).

### 2.2 Plot survey and soil nutrients (N and P) analysis

Within each plot, all trees with a diameter at breast height (DBH, 1.3 m above the ground) greater than 1 cm were recorded and identified in July 2013. Tree richness (number of tree species within a plot) and effective number of species (calculated as *e* ^*H*′^, with $H^{\prime }=-\sum f_{i}\text{Ln}f_{i}$, where *f*_*i*_ is the proportion of stems in a plot belonging to the *i*th species; Chao, Chiu, & Jost, 2010; Hill, 1973) were determined for each plot. As the tree abundance varies between plots, the tree richness of each plot was rarefied (rarefied tree richness, *R*_TR_) to the smallest sample size using the community ecology R package “vegan” (Oksanen et al., 2015; R Core Team, 2015). In each plot, we sampled 500 g of soil from each of the four corners from the 1 to 10 cm depth below the litter layer in May 2013. A microdiffusion method was used to determine alkali‐hydrolyzable nitrogen (AN) in the soil, and extractable phosphorus (EP) was extracted with solution containing 0.03 mol/L NH_4_F and 0.025 mol/L HCl and estimated colorimetrically following the protocol as described in Hu et al. (2012).

### 2.3 Community phylogenetic reconstruction and phylogenetic diversity

**Figure 2:**
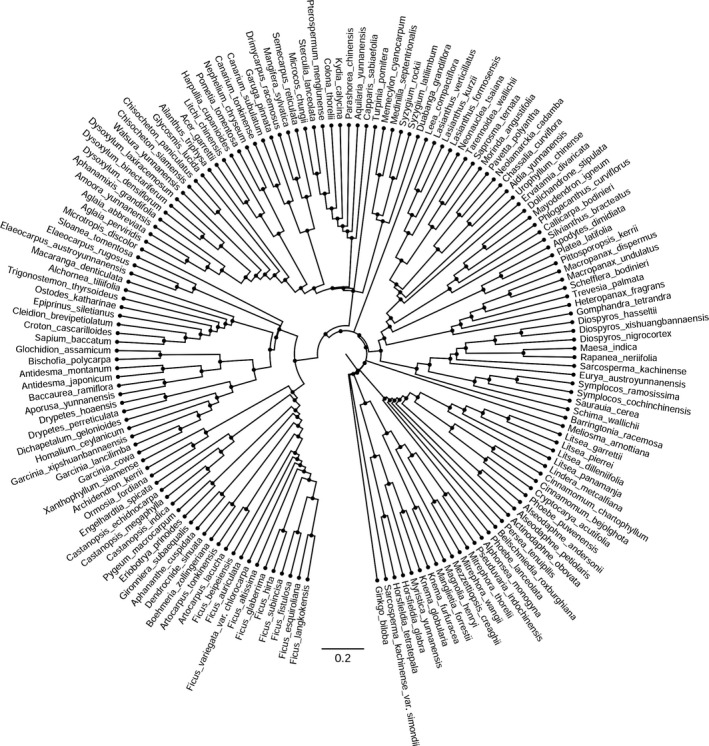
The community phylogeny of 156 tree species recorded in the 39 plots. The community phylogeny was constructed based on a maximum likelihood analysis of *rbc*L, *mat*K, *psb*A‐*trn*H, and ITS sequence data with APG III as a constraint tree

We used the molecular phylogeny of 156 taxa recorded within our plots for community phylogenetic structure analysis. The species recorded are listed in Appendix S1. The phylogeny was assembled using RAxML (Stamatakis, 2006) based on the DNA barcodes *rbc*L, *mat*K, *trn*H–*psb*A, and ITS (the original sequences have been reported in Huang, Ci, Conran, & Li, 2015). A semiparametric method based on a penalized likelihood in the R statistical software package “ape” was used to generate an ultrametric phylogenetic tree (Paradis, Claude, & Strimmer, 2004; R Core Team, 2015; Figure 2, Appendix S2). The mean pairwise phylogenetic distances (MPD) among individual tree species within each plot (400 m^2^; *n* = 39) were calculated using the R package “picante” (Kembel et al., 2010; R Core Team, 2015). The MPD was assumed to reflect the phylogenetic structure across the entire phylogeny (Webb, 2000). For the comparison of communities in the plots, the observed value of the MPD was standardized as follows: standardized effect size (SES) = (observed value − mean of 9,999 randomized values)/standard deviation, where the randomized value was calculated using the null model “taxa.labels” (Kembel et al., 2010) and the MPD was not weighted by species abundance (Stevens et al., 2012). The net relatedness index (NRI) was calculated by multiplying SES by negative one; a positive NRI value for a particular community indicated phylogenetic clustering, whereas a negative value indicated phylogenetic overdispersion (Kembel et al., 2010; Webb, 2000).

Based on the molecular phylogeny constructed, the rarefied Faith's phylogenetic diversity (PD, rarefied to the smallest sample size of 84; Faith, 1992; Nipperess & Matsen, 2013; R Core Team, 2015) and the phylogenetic diversity based on Hill numbers (*q* = 1; Chao et al., 2010; Marcon & Herault, 2015; R Core Team, 2015) were calculated for each plot.

### 2.4 Statistical analyses

The mean and coefficient of variation (CV = *SD*/*M*,* SD* = standard deviation, *M *= mean) of AN and EP were calculated to represent the availability and heterogeneity of these nutrients within each plot (Baer, Blair, Collins, & Knapp, 2004; Douda et al., 2012; Holl et al., 2013). The Shapiro–Wilk test was first implemented to evaluate the normal distribution of all variables; all the soil variables within each plot were log‐transformed to promote normality. Pearson correlations were used to explore the relations among soil nutrient (N and P) availability and heterogeneity, tree species diversity, and NRI across the plots. Both Shapiro–Wilk tests and Pearson correlations were implemented using the SPSS 16.0 statistical software package (SPSS Inc, Chicago, IL, USA). The significance was determined at *p *<* *.05.

## 3 Results

### 3.1 Plot characteristics

The content and CV of AN and EP in the 39 plots was highly variable with AN varying between 123.25 and 240.25 mg/kg (CV: 0.02–0.35), and EP ranging from 2.43 to 23.4 mg/kg (CV: 0.16–1.13). The content and CV of EP was significantly correlated within the plots (*r *=* *.525, *p *=* *.001) while the content and CV of AN was not (*r *=* *−.214, *p *=* *.190; Figure S3). A total of 167 tree species (with DBH > 1 cm) were identified from the 39 plots (Appendix S1) with an average of 36 tree species in each plot, ranging from 25 to 47. The rarefied tree richness ranged from 19 to 34 in the plots. The NRI in the 39 plots ranged from −1.49 (overdispersed) to 2.75 (clustered), with an average of −0.075.

### 3.2 Soil nutrient (N and P) heterogeneity and tree species diversity

**Figure 3:**
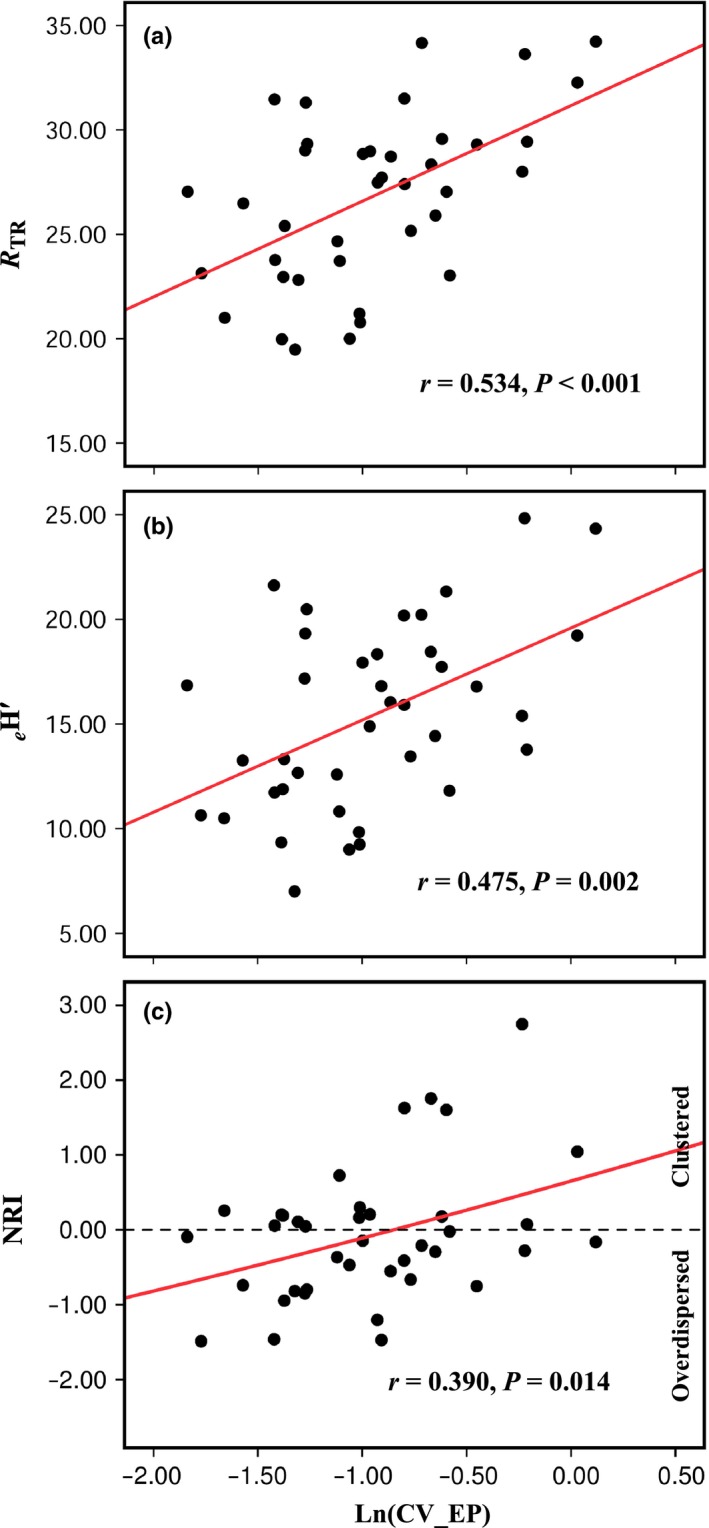
Effects of soil phosphorus heterogeneity on tree species diversity and community phylogenetic structure in the Xishuangbanna tropical seasonal rainforest in southwestern China. *R*_TR_, rarefied tree richness, rarefied to the smallest sample size of 84; *e*^*H*^′, effective number of species, with *H*′ as the Shannon–Wiener index; NRI, net relatedness index; CV_EP, the coefficient of variation of EP, Ln‐transformed; see section 2

**Figure 4:**
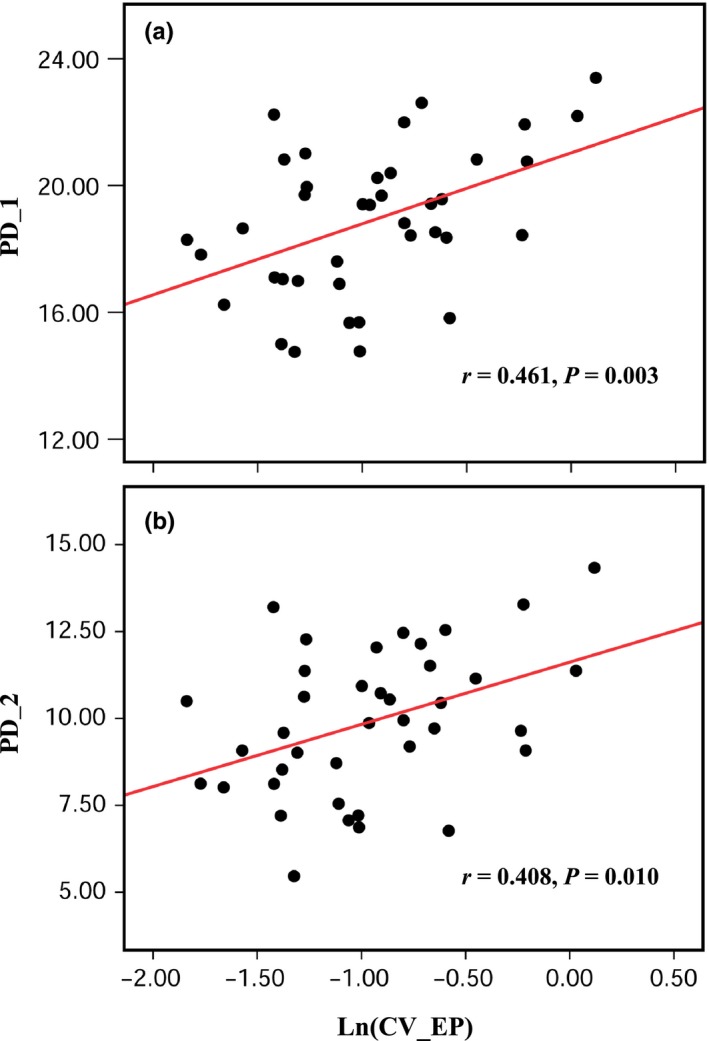
The increase in soil phosphorus heterogeneity promotes phylogenetic diversity of tree species within the community. PD_1, Faith's phylogenetic diversity, rarefied to the smallest sample size of 84; PD_2, phylogenetic diversity based on Hill numbers (*q* = 1); see section 2

Both AN and EP were not correlated with tree species diversity within the plots (AN and *R*_TR_: *r *=* *.087, *p *=* *.597; AN and *e* ^*H*′^: *r *=* *−.072, *p *=* *.665; EP and *R*_TR_: *r *=* *.086, *p *=* *.604; EP and *e* ^*H*′^: *r *=* *.049, *p *=* *.767; Figure S4). The CV of EP and tree species diversity within the plots was positively correlated (CV of EP and *R*_TR_: *r *=* *.534, *p *<* *.001; CV of EP and *e* ^*H*′^: *r *=* *.475, *p *=* *.002; Figure 3a,b). However, there was no correlation between CV of AN and tree species diversity (CV of AN and *R*_TR_: *r *=* *.137, *p *=* *.405; CV of AN and *e* ^*H*′^: *r *=* *.228, *p *=* *.162; Figure S5a,b). Soil phosphorus heterogeneity and the phylogenetic diversity within the plots were significantly correlated (Figure 4), while there was no correlation between the soil nitrogen heterogeneity and the phylogenetic diversity within the plots (Figure S6).

### 3.3 Soil nutrient (N and P) heterogeneity and community phylogenetic structure

The CV of EP and NRI was positively correlated (*r *=* *.390; *p *=* *.014; Figure 3c) while the CV of AN and NRI was not (*r *=* *.212; *p *=* *.194; Figure S5c). NRI and tree species richness within the plots was not correlated (*r *=* *.006; *p *=* *.970).

## 4 Discussion

### 4.1 Soil nutrient (N and P) heterogeneity and tree species diversity

It has long been hypothesized that local environmental heterogeneity significantly affects the distribution and diversity of plants in the tropics (Ricklefs, 1977), while a direct positive correlation between environmental heterogeneity and plant diversity in the tropics has yet to be demonstrated (Holl et al., 2013). On the basis of the analysis from 39 plots within the Xishuangbanna tropical seasonal rainforest, we revealed that soil phosphorus heterogeneity significantly promoted tree species diversity which provided evidence that deterministic and not neutral processes dominated in the assembly of the Xishuangbanna tropical seasonal rainforest (Yang et al., 2014, 2015).

Resource availability and heterogeneity usually covary and confound the effects of resource heterogeneity on species diversity (Lundholm, 2009; Stevens & Carson, 2002). In this study, we revealed that soil phosphorus heterogeneity but not the availability significantly affects tree species diversity within community although the soil phosphorus heterogeneity and availability are also correlated (Figure S3b). Our previous study suggested that phosphorus availability significantly affects tree species diversity in the Xishuangbanna tropical seasonal rainforest (Xu et al., 2016). Compared with 40 × 40 m plot in the previous study, 20 × 20 m plot was set in the current study. It has been suggested that competitive exclusion is more apparent at smaller spatial scales while environmental filtering is more apparent at medium‐to‐large scales (Swenson, Enquist, Thompson, & Zimmerman, 2007). It is likely that increasing resource heterogeneity at small scales moderates the competitive exclusion among species and promotes species coexistence. With the increases in scale, competitive exclusion among species could be relaxed and increasing resource availability may facilitate tree species passing through the environmental filters (e.g., low phosphorus availability) and promote species diversity (Xu et al., 2016).

Neither soil nitrogen availability nor its heterogeneity was correlated with tree species diversity within the plots. It has been suggested that nitrogen levels in the tropical soils are relatively high (Huston, 1980). When the availability of a particular resource within the community is high, the effect of heterogeneity of such resource on species diversity is usually relatively low, as predicted in resource competition theory (Tilman, 1982). Therefore, an increase in soil nitrogen availability or heterogeneity may not affect tree species diversity because it is not a limiting nutrient.

### 4.2 Soil nutrient (N and P) heterogeneity and community phylogenetic structure

Many studies, including the current one, revealed a positive correlation between heterogeneity and diversity (e.g., Stein et al., 2014), while the mechanism underlying the influence of heterogeneity on diversity is unclear. Community phylogenetic analysis provides opportunities to examine possible mechanisms (Brown, 2012; Cavender‐Bares et al., 2009; Joly et al., 2014; Swenson et al., 2007; Willis et al., 2010). Stevens et al. (2012) provided the first example on the study of how environmental heterogeneity affects species diversity within the community. These authors have found a significant positive correlation between rodent species diversity and food heterogeneity within a community. Their further community phylogenetic analyses have revealed that increased food heterogeneity promoted phylogenetic clustering as more closely related species coexist within the community. In our study, the soil nitrogen heterogeneity within the plots was neither related to the tree species diversity nor to community phylogenetic structure (Figure S5). However, we identified a significant positive correlation between soil phosphorus heterogeneity and tree species diversity in the Xishuangbanna tropical seasonal rainforest (Figure 3a,b), and further community phylogenetic analysis indicated that the trees in plots with low soil phosphorus heterogeneity were phylogenetically overdispersed, whereas those in plots with high heterogeneity were clustered (Figure 3c).

The competition among trees in a homogeneous environment may lead to a more dispersed community because closely related trees, sharing similar ecological requirements, would be eliminated from the community; however, recent studies have indicated that increased competitive exclusion among species may also eliminate the less related species and lead to a clustered community if competitive ability differences among species is more important than niche differences and positively correlated with phylogenetic distances (Godoy, Kraft, & Levine, 2014; Mayfield & Levine, 2010). In our study, an increase in soil phosphorus heterogeneity significantly promoted tree species diversity within the plots and the phylogenetic structure of trees in plots with low soil phosphorus heterogeneity was phylogenetically overdispersed, but tended to be clustered with the increase in soil phosphorus heterogeneity. Therefore, in the less heterogeneous communities, interspecific competition could eliminate the more closely related tree species, which led to a phylogenetically overdispersed community; however, with an increase in soil phosphorus heterogeneity, the more closely related tree species could coexist within the community because the interspecific competition was moderated (Stevens et al., 2012). It is worth noting that we only collected four soil samples in each plot, while the results of the significant correlation between phosphorus heterogeneity and tree diversity indicate that tree species diversity within the plots increased substantially along the gradient of phosphorus heterogeneity. Future research would benefit from more extensive sampling within the plots.

## Conflict of Interests

The authors declare no conflict of interests.
