# Supplementary material for: Soil phosphorus heterogeneity promotes tree species diversity and phylogenetic clustering in a tropical seasonal rainforest
Source: Ecol Evol. 2016 Nov 16;6(24):8719–26. doi: 10.1002/ece3.2529 (PMC5192821; doi:10.1002/ece3.2529)
Supplement: Supplementary file 1 [file ECE3-6-8719-s001.docx]

**Supplementary information**

**
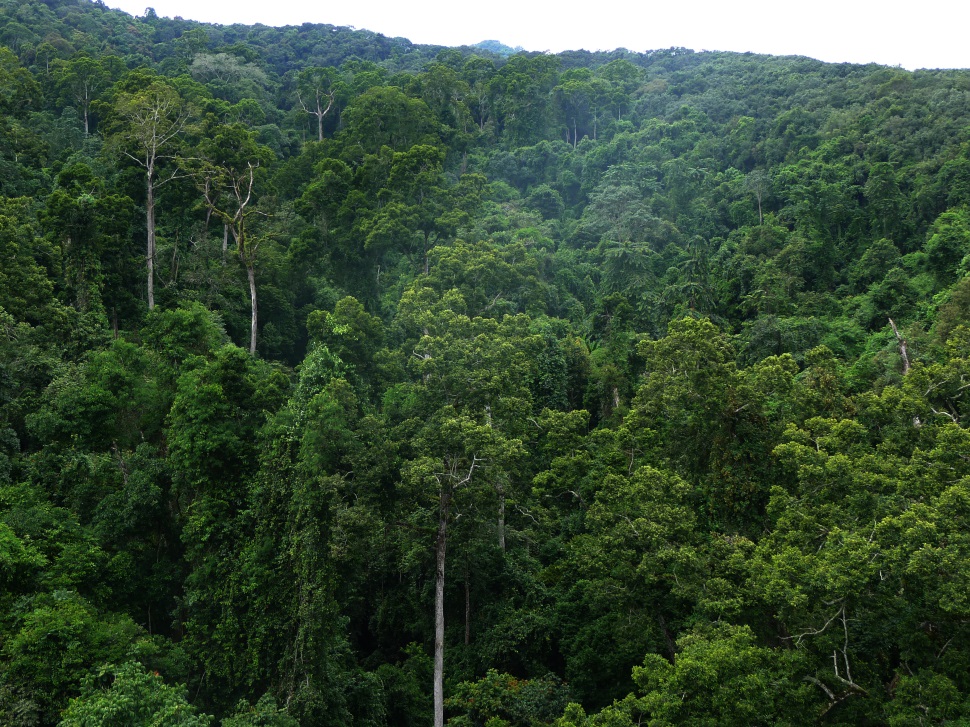
**

**Figure S1.** The Xishuangbanna tropical seasonal rainforest in southwestern China.


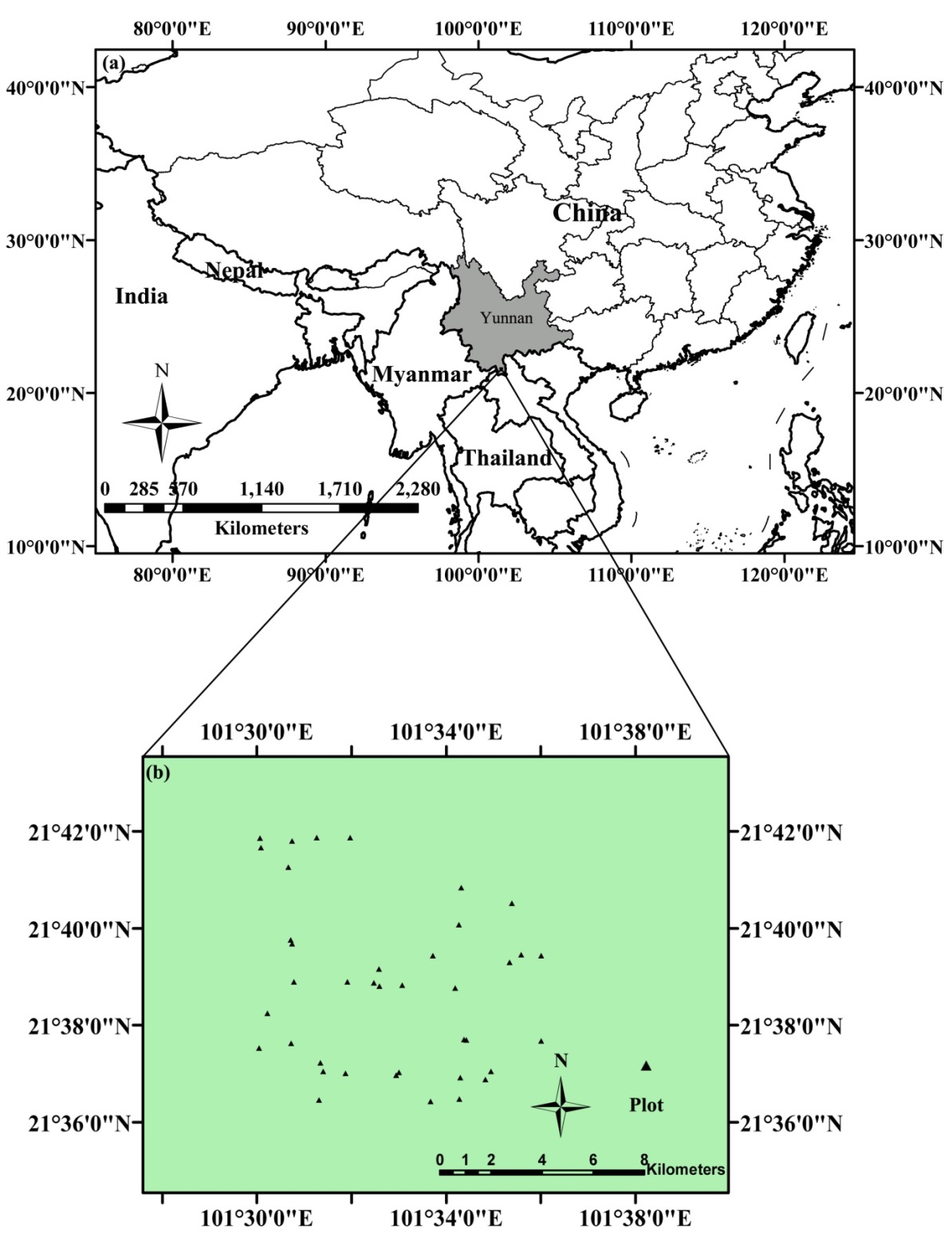


**Figure S2**. Plot locations in the Xishuangbanna tropical seasonal rainforest.


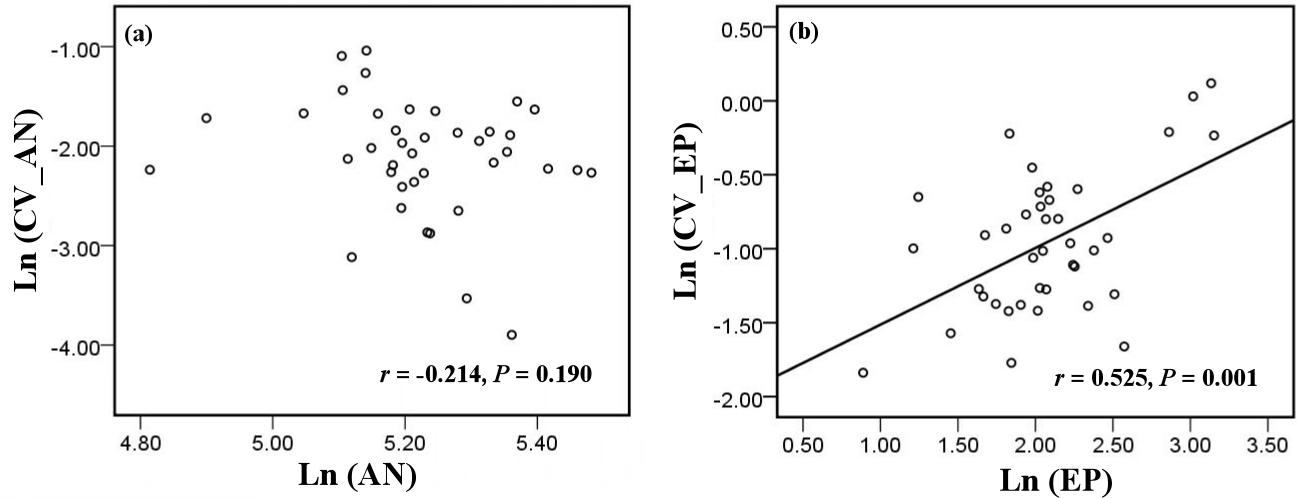


**Figure S3**. Correlations between soil nutrients (AN and EP) availability and their heterogeneity within each plot.


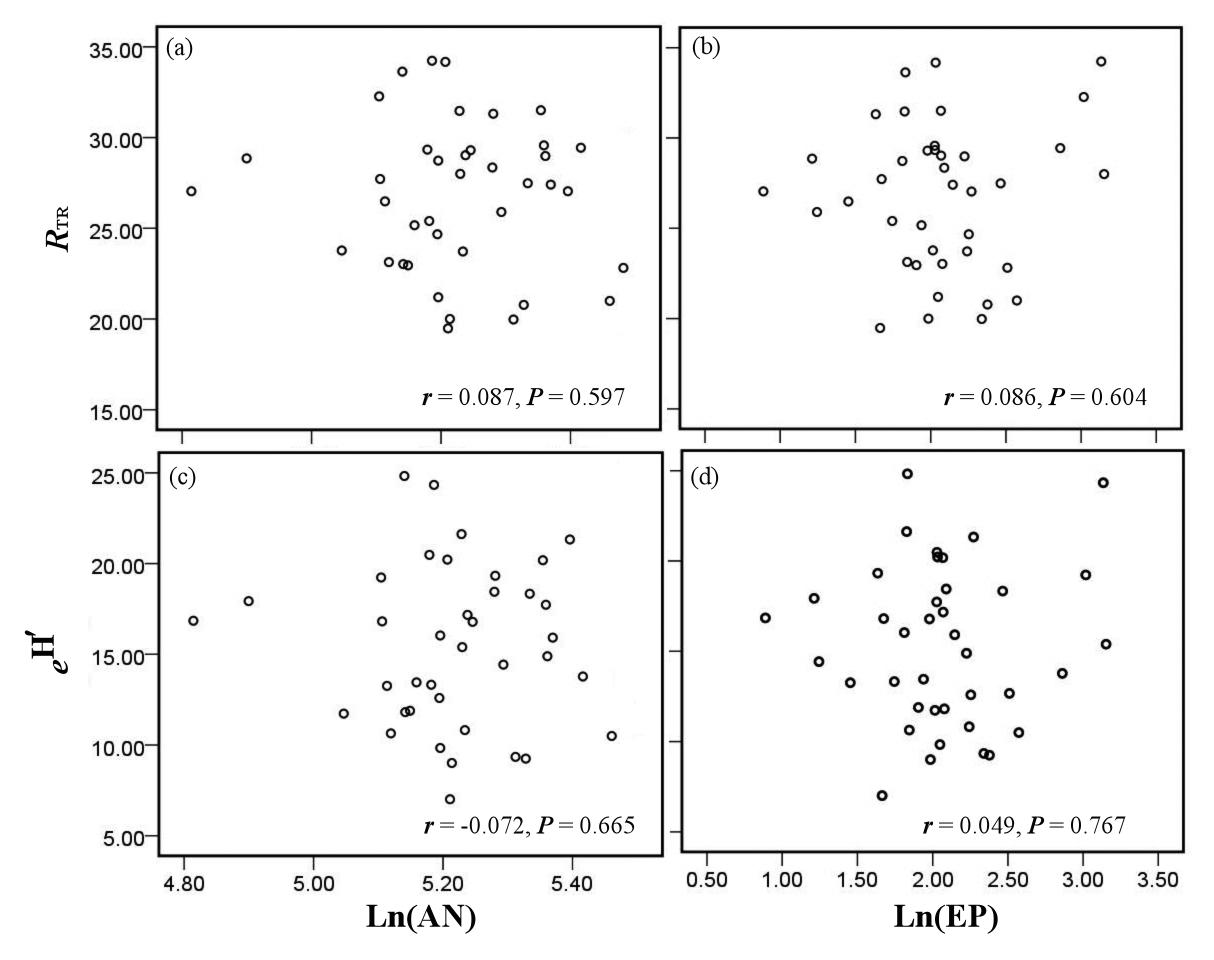


**Figure S4**. Correlations between soil nutrients (AN and EP) availability and tree species diversity within each plot. *R*_TR_, rarefied tree richness, rarefied to the smallest sample size of 84; *e*^H′^, effective number of species, with H′ as the Shannon-Wiener index.

**
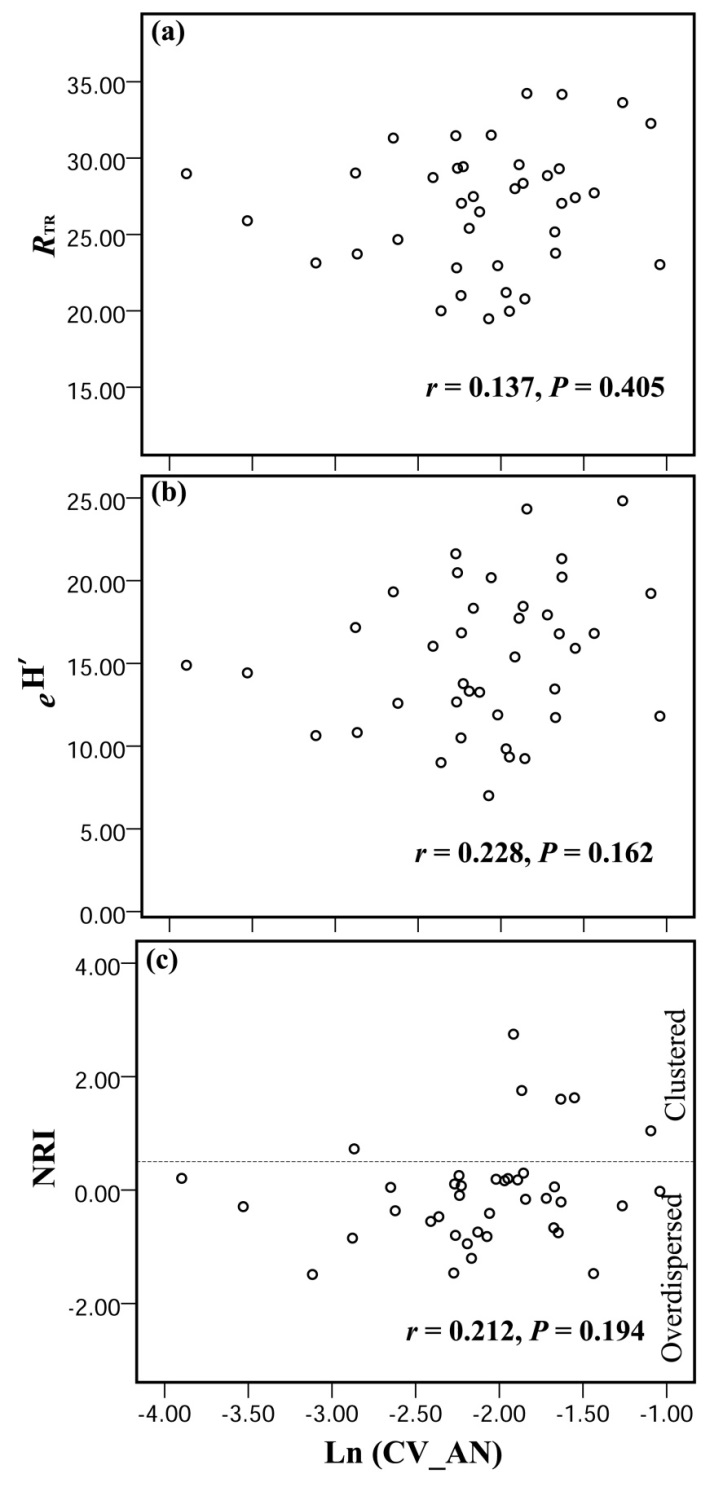
Figure S5.** Correlations between soil nitrogen heterogeneity, tree species diversity and community phylogenetic structure in the Xishuangbanna tropical seasonal rainforest in southwestern China. NRI, net relatedness index; CV_AN, the coefficient of variation of AN, Ln-transformed; see Methods.

**
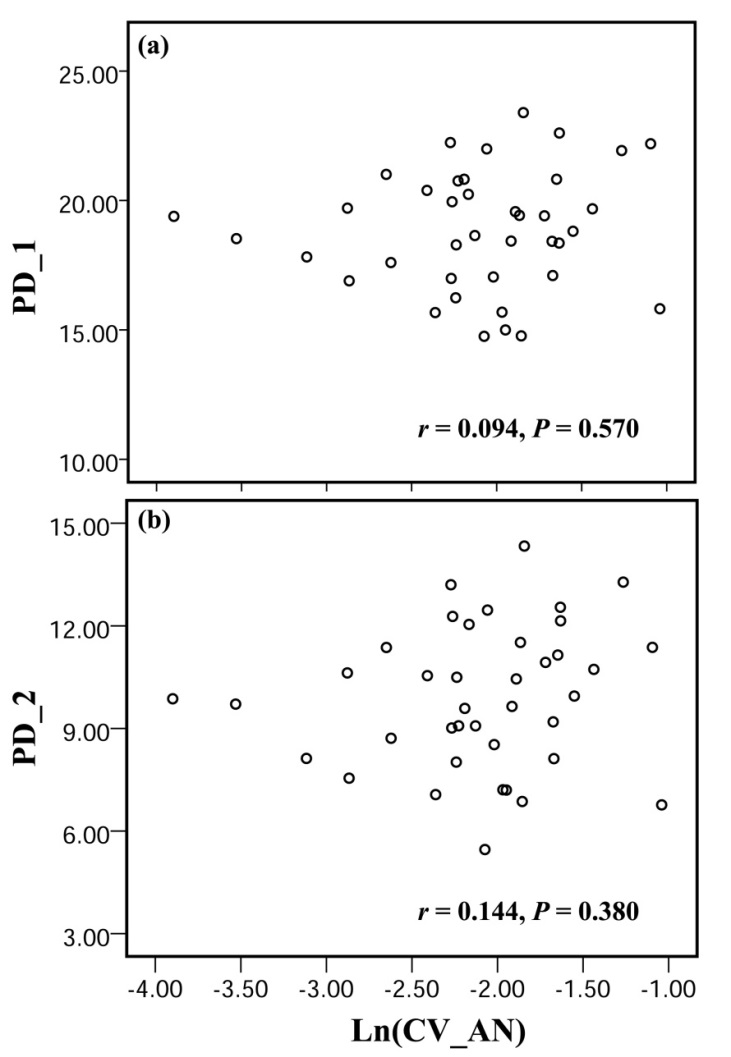
**

**Figure S6.** Correlations between soil nitrogen heterogeneity and phylogenetic diversity in the Xishuangbanna tropical seasonal rainforest in southwestern China. PD_1, Faith’s phylogenetic diversity, rarefied to the smallest sample size of 84; PD_2, phylogenetic diversity based on Hill numbers (q = 1); see Methods.

**Appendix S1.** List of trees with a DBH>1 cm recorded in the 39 plots. The trees recorded in the plots that are not used in community phylogenetic reconstruction because of the absence of DNA barcode data are shown in bold. *Ficus chlorocarpa* and *Ficus sp. 1* were replaced by the closely related tree species *Ficus altissima* and *Ficus variegate var. chlorocarpa* during the community phylogenetic reconstruction.

| Code | Genus | Species |
| --- | --- | --- |
| 1 | *Acer* | *garrettii* |
| 2 | *Actinodaphne* | *obovata* |
| 3 | *Aglaia* | *abbreviata* |
| 4 | *Aglaia* | *perviridis* |
| 5 | *Aidia* | *yunnanensis* |
| 6 | *Ailanthus* | *triphysa* |
| 7 | *Alchornea* | *tiliifolia* |
| 8 | *Alphonsea* | *monogyna* |
| 9 | *Alseodaphne* | *andersonii* |
| 10 | *Alseodaphne* | *petiolaris* |
| 11 | ***Alsophila*** | ***spinulosa*** |
| 12 | *Amoora* | *yunnanensis* |
| 13 | *Antidesma* | *japonicum* |
| 14 | *Antidesma* | *montanum* |
| 15 | *Aphanamixis* | *grandifolia* |
| 16 | *Aphananthe* | *cuspidata* |
| 17 | *Apodytes* | *dimidiata* |
| 18 | *Aporusa* | *yunnanensis* |
| 19 | *Aquilaria* | *yunnanensis* |
| 20 | ***Ardisia*** | ***tenera*** |
| 21 | *Artocarpus* | *lacucha* |
| 22 | *Artocarpus* | *tonkinensis* |
| 23 | *Baccaurea* | *ramiflora* |
| 24 | ***Baphicacanthus*** | ***cusia*** |
| 25 | *Barringtonia* | *racemosa* |
| 26 | *Beilschmiedia* | *roxburghiana* |
| 27 | *Bischofia* | *polycarpa* |
| 28 | *Boehmeria* | *zollingeriana* |
| 29 | *Callicarpa* | *bodinieri* |
| 30 | *Canarium* | *subulatum* |
| 31 | *Canarium* | *tonkinense* |
| 32 | *Capparis* | *sabiaefolia* |
| 33 | ***Casearia*** | ***balansae*** |
| 34 | *Castanopsis* | *echidnocarpa* |
| 35 | *Castanopsis* | *indica* |
| 36 | *Castanopsis* | *megaphylla* |
| 37 | *Chassalia* | *curviflora* |
| 38 | *Chisocheton* | *paniculatus* |
| 39 | *Chisocheton* | *siamensis* |
| 40 | *Cinnamomum* | *bejolghota* |
| 41 | *Cinnamomum* | *chartophyllum* |
| 42 | *Cleidion* | *brevipetiolatum* |
| 43 | *Colona* | *thorelii* |
| 44 | *Croton* | *cascarilloides* |
| 45 | *Cryptocarya* | *acutifolia* |
| 46 | *Archidendron* | *kerrii* |
| 47 | ***Dalbergia*** | ***sp1*** |
| 48 | *Dendrocnide* | *sinuata* |
| 49 | *Dichapetalum* | *gelonioides* |
| 50 | *Diospyros* | *xishuangbannaensis* |
| 51 | *Diospyros* | *hasseltii* |
| 52 | *Diospyros* | *nigrocortex* |
| 53 | *Dolichandrone* | *stipulata* |
| 54 | *Drimycarpus* | *racemosus* |
| 55 | *Drypetes* | *hoaensis* |
| 56 | *Drypetes* | *perreticulata* |
| 57 | *Duabanga* | *grandiflora* |
| 58 | *Dysoxylum* | *binectariferum* |
| 59 | *Dysoxylum* | *densiflorum* |
| 60 | *Dysoxylum* | *laxiracemosum* |
| 61 | *Elaeocarpus* | *austroyunnanensis* |
| 62 | *Elaeocarpus* | *rugosus* |
| 63 | *Engelhardtia* | *spicata* |
| 64 | *Epiprinus* | *siletianus* |
| 65 | *Eriobotrya* | *prinoides* |
| 66 | *Ervatamia* | *divaricata* |
| 67 | *Eurya* | *austroyunnanensis* |
| 68 | *Ficus* | *beipeiensis* |
| 69 | *Ficus* | *hirta* |
| 70 | *Ficus* | *auriculata* |
| 71 | ***Ficus*** | ***chlorlcarpa*** |
| 72 | *Ficus* | *esquiroliana* |
| 73 | *Ficus* | *fistulosa* |
| 74 | *Ficus* | *glaberrima* |
| 75 | *Ficus* | *langkokensis* |
| 76 | ***Ficus*** | ***sp1*** |
| 77 | *Ficus* | *subincisa* |
| 78 | *Garcinia* | *cowa* |
| 79 | *Garcinia* | *lancilimba* |
| 80 | *Garcinia* | *xipshuanbannaensis* |
| 81 | *Garuga* | *pinnata* |
| 82 | *Gironniera* | *subaequalis* |
| 83 | *Glochidion* | *assamicum* |
| 84 | *Glycosmis* | *lucida* |
| 85 | *Gomphandra* | *tetrandra* |
| 86 | *Harpullia* | *cupanioides* |
| 87 | *Heteropanax* | *fragrans* |
| 88 | *Homalium* | *ceylanicum* |
| 89 | *Horsfieldia* | *glabra* |
| 90 | *Horsfieldia* | *tetratepala* |
| 91 | ***Ixora*** | ***amplexicaulis*** |
| 92 | *Knema* | *furfuracea* |
| 93 | *Knema* | *globularia* |
| 94 | *Kydia* | *calycina* |
| 95 | *Lasianthus* | *verticillatus* |
| 96 | *Lasianthus* | *formosensis* |
| 97 | *Lasianthus* | *kurzii* |
| 98 | *Leea* | *compactiflora* |
| 99 | *Lindera* | *metcalfiana* |
| 100 | *Litchi* | *chinensis* |
| 101 | ***Lithocarpus*** | ***craibianus*** |
| 102 | ***Litsea*** | ***balansae*** |
| 103 | *Litsea* | *dilleniifolia* |
| 104 | *Litsea* | *garrettii* |
| 105 | *Litsea* | *panamanja* |
| 106 | *Litsea* | *pierrei* |
| 107 | *Macaranga* | *denticulata* |
| 108 | *Macropanax* | *dispermus* |
| 109 | *Macropanax* | *undulatu* |
| 110 | *Maesa* | *indica* |
| 111 | *Magnolia* | *henryi* |
| 112 | *Mangifera* | *sylvatica* |
| 113 | *Manglietia* | *forrestii* |
| 114 | *Mayodendron* | *igneum* |
| 115 | *Medinilla* | *septentrionalis* |
| 116 | *Meliosma* | *arnottiana* |
| 117 | *Memecylon* | *cyanocarpum* |
| 118 | *Mezzettiopsis* | *creaghii* |
| 119 | *Microcos* | *chungii* |
| 120 | *Microtropis* | *discolor* |
| 121 | *Mitrephora* | *thorelii* |
| 122 | *Mitrephora* | *wangii* |
| 123 | *Morinda* | *angustifolia* |
| 124 | *Myristica* | *yunnanensis* |
| 125 | *Neolamarckia* | *cadamba* |
| 126 | *Neonauclea* | *tsaiana* |
| 127 | *Nephelium* | *chryseum* |
| 128 | ***Nothapodytes*** | ***foetida*** |
| 129 | *Ormosia* | *fordiana* |
| 130 | *Ostodes* | *katharinae* |
| 131 | ***Pandanus*** | ***tectorius*** |
| 132 | *Parashorea* | *chinensis* |
| 133 | *Pavetta* | *polyantha* |
| 134 | *Persea* | *tenuipilis* |
| 135 | *Phlogacanthus* | *curviflorus* |
| 136 | *Phoebe* | *lanceolata* |
| 137 | *Phoebe* | *puwenensis* |
| 138 | *Pittosporopsis* | *kerrii* |
| 139 | *Platea* | *latifolia* |
| 140 | *Pometia* | *tomentosa* |
| 141 | *Pseuduvaria* | *indochinensis* |
| 142 | *Pterospermum* | *menglunense* |
| 143 | *Pygeum* | *macrocarpum* |
| 144 | *Rapanea* | *neriifolia* |
| 145 | *Sapium* | *baccatum* |
| 146 | *Saprosma* | *ternata* |
| 147 | *Sarcosperma* | *kachinense* |
| 148 | *Sarcosperma* | *kachinense_var. simondii* |
| 149 | *Saurauia* | *cerea* |
| 150 | *Schefflera* | *bodinieri* |
| 151 | *Schima* | *wallichii* |
| 152 | *Semecarpus* | *reticulata* |
| 153 | *Silvianthus* | *bracteatus* |
| 154 | *Sloanea* | *tomentosa* |
| 155 | *Sterculia* | *lanceolata* |
| 156 | *Symplocos* | *cochinchinensis* |
| 157 | *Symplocos* | *ramosissima* |
| 158 | *Syzygium* | *latilimbum* |
| 159 | *Syzygium* | *rockii* |
| 160 | *Tarennoidea* | *wallichii* |
| 161 | *Trevesia* | *palmata* |
| 162 | *Trigonostemon* | *thyrsoideus* |
| 163 | *Turpinia* | *pomifera* |
| 164 | ***Ulmus*** | ***lanceaefolia*** |
| 165 | *Urophyllum* | *chinense* |
| 166 | *Walsura* | *yunnanensis* |
| 167 | *Xanthophyllum* | *siamense* |

**Appendix S2.** Sequence alignment and assembly of the DNA barcodes within this study.

The rbcL marker was aligned using ClustalW program (Thompson *et al*. 1994) with the standard parameters. The matK marker was aligned using back translation through the program transAlign (Bininda-Edmonds 2005). Because the ITS sequences were more difficult to align, we used the Simultaneous Alignment and Tree Estimation (SATé) for global multiple alignment (http://phylo.bio.ku.edu/software/sate/sate.html) (Liu *et al*. 2012). The trnH–psbA marker was highly variable and could not be handled with a global multiple sequence alignment, so we conducted a family-based alignment using ClustalW (Thompson *et al*. 1994) and then created a supermatrix by concatenating them with the aligned sequences of the other markers using Geneious 6.1.8 (Biomatters, Inc., http://www.geneious.com).

**References**

Bininda-Edmonds ORP (2005) transAlign: Using amino acids to facilitate the multiple alignment of protein-coding DNA sequences. *BMC Bioinformatics* **6**:156.

Liu K, Warnow TJ, Holder MT, Nelesen SM, Yu J, Stamatakis AP, et al. (2012) SATe-II: very fast and accurate simultaneous estimation of mul- tiple sequence alignments and phylogenetic trees. *Systematic Biology* **61**: 90–106.

Thompson JD, Higgins DG, Gibson TJ (1994) CLUSTAL W: improving the sensitivity of progressive multiple sequence alignment through sequence weighting, position specific gap penalties and weight matrix choice. *Nucleic Acids Research* **22**: 4673–4680.
